# Effect of end-tidal CO_2_ clamping on cerebrovascular function, oxygenation, and performance during 15-km time trial cycling in severe normobaric hypoxia: the role of cerebral O_2_ delivery

**DOI:** 10.1002/phy2.66

**Published:** 2013-08-28

**Authors:** Jui-Lin Fan, Nicolas Bourdillon, Bengt Kayser

**Affiliations:** 1 Institute of Sports Sciences, Faculty of Biology and Medicine, University of LausanneLausanne, Switzerland; 2 Lemanic Doctoral School of Neuroscience, University of LausanneLausanne, Switzerland

**Keywords:** CO_2_ clamping, exercise, hypoxia, ventilatory control

## Abstract

During heavy exercise, hyperventilation-induced hypocapnia leads to cerebral vasoconstriction, resulting in a reduction in cerebral blood flow (CBF). A reduction in CBF would impair cerebral O_2_ delivery and potentially account for reduced exercise performance in hypoxia. We tested the hypothesis that end-tidal Pco_2_ (PETCO_2_) clamping in hypoxic exercise would prevent the hypocapnia-induced reduction in CBF during heavy exercise, thus improving exercise performance. We measured PETCO_2_, middle cerebral artery velocity (MCAv; index of CBF), prefrontal cerebral cortex oxygenation (cerebral O_2_Hb; index of cerebral oxygenation), cerebral O_2_ delivery (DO_2_), and leg muscle oxygenation (muscle O_2_Hb) in 10 healthy men (age 27 ± 7 years; VO_2_max 63.3 ± 6.6 mL/kg/min; mean ± SD) during simulated 15-km time trial cycling (TT) in normoxia and hypoxia (FIO_2_ = 0.10) with and without CO_2_ clamping. During exercise, hypoxia elevated MCAv and lowered cerebral O_2_Hb, cerebral DO_2_, and muscle O_2_Hb (*P* < 0.001). CO_2_ clamping elevated PETCO_2_ and MCAv during exercise in both normoxic and hypoxic conditions (*P* < 0.001 and *P* = 0.024), but had no effect on either cerebral and muscle O_2_Hb (*P* = 0.118 and *P* = 0.124). Nevertheless, CO_2_ clamping elevated cerebral DO_2_ during TT in both normoxic and hypoxic conditions (*P* < 0.001). CO_2_ clamping restored cerebral DO_2_ to normoxic values during TT in hypoxia and tended to have a greater effect on TT performance in hypoxia compared to normoxia (*P* = 0.097). However, post hoc analysis revealed no effect of CO_2_ clamping on TT performance either in normoxia (*P* = 0.588) or in hypoxia (*P* = 0.108). Our findings confirm that the hyperventilation-induced hypocapnia and the subsequent drop in cerebral oxygenation are unlikely to be the cause of the reduced endurance exercise performance in hypoxia.

## Introduction

Altitude hypoxia represents a formidable environmental challenge to the human organism. Hypoxia limits oxygen transport from the air to the muscle mitochondria, which compromises aerobic capacity (Calbet and Lundby 2009). Exercise capacity is a vital determinant of a population's ability to thrive at high altitude (Curran et al. 1998), and permanent residence above 5000 m poses problems for mining and other human endeavor. Despite more than a century of research on the detrimental effects of hypoxia on exercise performance, the exact underlying mechanisms remain poorly understood [see (Amann and Kayser 2009; Verges et al. 2012) for reviews].

Even though the cessation of maximal exercise, or the reduction in exercise intensity, when fatiguing, imply reduced motor recruitment by the central nervous system, the mechanisms that lead to the derecruitment of active muscle remain elusive. During normoxia and moderate hypoxia, leg muscle afferents appear to play an important role (Amann 2006; Amann and Dempsey 2007; Amann et al. 2011), whereas cerebral tissue oxygenation may play a more pivotal role under severe hypoxic conditions (Kjaer et al. 1999; Amann et al. 2006; Amann and Dempsey 2007; Subudhi et al. 2007a*,* 2009; Rasmussen et al. 2010; Vogiatzis et al. 2011). At rest, the brain protects itself against hypoxia by increasing cerebral blood flow (CBF) to compensate for the decreased arterial oxygen tension (Cohen et al. 1967). But during high-intensity exercise, hyperventilation-induced hypocapnia leads to cerebral vasoconstriction, which counteracts the hypoxia-induced vasodilation, thereby lowering CBF and cerebral oxygenation (Jorgensen et al. 1992a; Madsen et al. 1993; Ide et al. 1999; Subudhi et al. 2007b). As hypoxia per se enhances ventilatory drive, this would further exacerbate the hyperventilation-induced hypocapnia during heavy exercise and further lower CBF and cerebral oxygenation. It was proposed that this reduction in cerebral oxygenation may account for the reduced performance during heavy exercise in severe hypoxia (Amann and Dempsey 2007; Nybo and Rasmussen 2007; Amann and Kayser 2009; Subudhi et al. 2009; Rasmussen et al. 2010). In support, reduced cerebral oxygenation has been shown to coincide with both reduced cortical motor output (Subudhi et al. 2009) and increased cerebral metabolism (Rasmussen et al. 2010).

Contrary to this hypothesis, Subudhi et al. (2011) recently found exercise capacity to be *reduced* when they prevented the normal hyperventilation-induced hypocapnia with end-tidal CO_2_ clamping during incremental cycling in hypobaric hypoxia (equivalent of 4875 m), despite elevated CBF and improved cerebral oxygenation. They attributed this finding to limiting factors such as increased respiratory muscle work and associated “steal” of blood flow, and an elevated perceived effort related to their experimental setup, which may have outweighed the benefits of elevated cerebral O_2_ delivery. Siebenmann et al. (2012) completed those observations by investigating, at a more moderate altitude (3454 m), the impact of clamping PETCO_2_ at 40 mmHg, on performance. Clamping increased middle cerebral artery velocity (MCAv), attenuated the decrease in cerebral oxygenation, but slightly decreased peak power output while it did not affect maximal oxygen uptake. Siebenmann et al. (2012) concluded that although hypocapnia decreases CBF and cerebral oxygenation, this does not limit maximum aerobic exercise capacity. In contrast, using a similar approach but in nonacclimatized subjects during incremental exercise to capacity in more severe hypoxia (equivalent of 5000 m), we were unable to improve CBF with inspired CO_2_, despite a greater degree of hypercapnia (Fan and Kayser 2013). We attributed these discrepant findings to the different acclimatization states of the subjects in the different studies (389 m vs. 1600 m resident) and the type of hypoxia used (normobaric vs. hypobaric). However, all the aforementioned studies only looked at aerobic capacity using incremental exercise tests until voluntary exhaustion, which have limited ecological validity (Currell and Jeukendrup 2008). It thus remains unclear whether preventing a reduction in CBF and cerebral oxygenation associated with hyperventilation-induced hypocapnia would improve performance during prolonged, submaximal exercise paradigms such as time trial cycling, which are more representative of usual human exercise behavior.

In this study, we therefore examined the effect of clamping partial pressure of end-tidal CO_2_ (PETCO_2_) on CBF and performance during a 15-km time trial cycling in severe normobaric hypoxia, as time trial protocols provide a more accurate simulation of physiological responses during actual competitions (Foster et al. 1993) and has been shown to correlate well with actual race performance (Palmer et al. 1996). We tested the hypothesis that by preventing hyperventilation-induced hypocapnia, we would increase CBF and cerebral oxygen delivery without altering muscle tissue oxygenation during high-intensity exercise, improving exercise performance during 15-km time trial cycling at a simulated altitude of 5000 m in nonacclimatized low-altitude residents.

## Methods

### Participants

A power calculation was employed using a difference worth detecting of a 5% improvement in 15-km time trial cycling performance based upon previous findings by Jeukendrup et al. (2008), along with unpublished data from our laboratory. A sample size of nine control volunteers was sufficient to give a power of 0.80 and an α of 0.05. Ten healthy males with a mean age of 27 ± 7 year (mean ± SD), a body mass index of 22.3 ± 1.3 kg/m^2^, VO_2_max of 63.3 ± 6.6 mL/min^/^kg, and maximal power output of 385 ± 30 W participated in this study. Participants were nonsmokers, had no previous history of cardiovascular, cerebrovascular, or respiratory disease and were not taking any medication. All participants were informed regarding the procedures of this study, and informed consent was given prior to participation. All the participants were residents of Geneva, Switzerland (<500 m), and were trained cyclists. The study was approved by the Research Ethical Committee of the University Hospitals of Geneva and conformed to the standards set by the *Declaration of Helsinki*.

### Experimental design

The participants visited the laboratory on five occasions. Following full familiarization, which included a VO_2_max test to assess the participant's aerobic fitness (visit one), the participants underwent four experimental trials (randomized, balanced order, and in single-blind fashion) which consisted of the following conditions: (i) control normoxia (389 m); (ii) normoxia with CO_2_ clamping; (iii) control normobaric hypoxia (FIO_2_ = 0.10; simulated altitude of 5000 m); and (iv) normobaric hypoxia with CO_2_ clamping. Before each experimental session, the participants were asked to abstain from caffeine for 12 h, and heavy exercise and alcohol for 24 h. Each experimental testing session comprised of 20-min instrumentation with the participants breathing room air while seated on a bicycle fitted to a Computrainer Pro Model trainer, calibrated according to the manufacturer's instructions (RacerMate, Seattle, WA). While breathing normal room air, 4-min baseline data were collected (room air baseline), the participants then performed a 5-min self-selected warmup exercise (heart rate <120 bpm). They were then switched to breathe from a modified gas mixing system (Altitrainer, SMTec, Nyon, Switzerland) followed by an additional 4-min resting baseline collection (condition baseline). The participants then did a 15-km time trial cycling as fast as possible. They were free to shift gears during the time trials, and constant feedback regarding the distance covered, but not speed, was provided on a computer screen (Computrainer™ 3D software version 3.0; Racermate). To prevent excessive thermal stress during the time trial exercise, two ventilators were placed ∼60 cm in front of the participants and wind velocity was adjusted according to cycling speed. Throughout each experimental session, the participants wore a nose clip and breathed through a mouthpiece attached to a low-resistance one-way nonrebreathing valve (Hans-Rudolph 2700, Kansas City, MO). The simulated normobaric hypoxia and CO_2_ clamping were achieved using the gas mixing system, which was attached to the inspiratory valve of the mouthpiece using a piece of large-bore low-resistance tubing. The device consists of a reservoir in which air and experimental gases are mixed and from which the participant inspires. This setup enables bleeding additional CO_2_ into the inspired gas mixture, thereby increasing the fraction of inspired CO_2_ (FICO_2_) while keeping the fraction of inspired O_2_ (FIO_2_) constant at either 0.21 (as in ambient air) or 0.10 (in Geneva the equivalent of an altitude of ∼5000 m) during normoxia and hypoxia conditions, respectively. PETCO_2_ clamping was achieved under feedback of on-screen PETCO_2_ (LabChart 7.2; ADInstruments, Colorado Springs, CO), continuously adapting FICO_2_ keeping PETCO_2_ to its target value (45 mmHg). The participants breathed through the same circuit in all four conditions and were unaware to what gas mixture they were breathing and naive to the exact rationale of the study. For each participant, the experiments were carried out at the same time of day under consistent laboratory conditions (temperature 21 ± 1°C, humidity 32 ± 5%, barometric pressure 726 ± 6 mmHg).

### Measurements

#### Respiratory variables

Gas exchange was monitored on a breath-by-breath basis (Medgraphics CPX, Loma Linda, CA) measuring flow at the mouth with a Pitot tube and the fractions of inspired and expired O_2_ and CO_2_ with fast responding gas analyzers (infrared and paramagnetic) integrated in the system. Ventilation (V'E) was derived from the flow signal and expressed in body temperature, pressure, saturated and per minute. The partial pressures of end-tidal O_2_ (PETO_2_) and CO_2_ (PETO_2_) were derived from the expired O_2_ and CO_2_ signals. Prior to each experimental session, the system was calibrated using a 3-L syringe (M9474; Medikro Oy, Finland) and precision gas mixtures of known O_2_ and CO_2_ concentrations (Carbogas AG, Gümligen, Switzerland).

#### Cerebrovascular and cardiovascular variables

Middle cerebral artery velocity (as an index of CBF) was measured bilaterally in the middle cerebral arteries using a 2-MHz pulsed Doppler ultrasound system (ST3; Spencer technology, Seattle, WA). The ultrasound probes were positioned over the temporal windows and held firmly in place with an adjustable headband (Marc 600 Head Frame; Spencer Technology). The signals were obtained by first locating the bifurcation of the middle and anterior cerebral artery; the angle and depth of insonation were then adjusted to obtain measurements from the MCA. The insonation depth and the velocity of MCA signals were recorded and compared to ensure within-subject repeatability of MCAv measurements between trials. In our hands, day-to-day reproducibility of MCAv has a coefficient of variation of <10%. The bilateral MCAv was averaged to represent global CBF during rest and exercise. Cerebral O_2_ delivery (cerebral DO_2_) was calculated using the equation: cerebral DO_2_ = mean MCAv × arterial oxygen content (CaO_2_, see below). Cerebral DO_2_ was normalized to the room air baseline values prior to 5-min warmup during each trial and expressed as %.

Cerebral oxygenation in the left prefrontal lobe was assessed by monitoring changes in oxy- (O_2_Hb), deoxy- (HHb), and delta (delta Hb: O_2_Hb–HHb) hemoglobin obtained with spatially resolved, continuous wave near-infrared spectroscopy (NIRS; Artinis Oxymon, MKIII, Zetten, the Netherlands). Source detector spacing was set at 4.1 cm and data obtained from the optode were used to calculate changes in O_2_Hb and HHb with a differential pathlength factor (DPF) calculated using the formula: DPF = 4.99 + 0.067 × (age^0.814^) (Duncan et al. 1995). Muscle oxygenation in the left vastus lateralis (∼15 cm proximal and 5 cm lateral to the superior border of the patella) was measured with an additional NIRS channel on the same instrument using a source detector spacing of 3.8 cm and DPF of 4.0 (Duncan et al. 1995). The cerebral and muscle O_2_Hb and HHb are expressed at absolute changes from room air baseline prior to the 5-min warmup period. Beat-to-beat mean arterial blood pressure (MAP) was monitored using finger plethysmography (Finometer® MIDI, Finapress Medical Systems, Amsterdam, the Netherlands). Peripheral O_2_ saturation (SpO_2_) was measured using earlobe pulse oximetry (Radical-7; Masimo Corporation, Irvine, CA).

#### Blood gas variables

Arterialized earlobe capillary blood samples were taken at rest and every 5 km during exercise (Mollard et al. 2010). Vasodilating cream was applied to the earlobe 5 min prior to the sampling (Decontractyl, Sanofi Aventis, France). A lancet was used to pierce the earlobe and 60-μL capillary tubes (MultiCap; Siemens Healthcare Diagnostics Inc, Tarrytown, UK) were used to collect the samples. All samples were analyzed immediately (<5 sec) using an arterial blood gas analyzing system (Rapidlab™ 248; Siemens Healthcare Diagnostics Inc) for arterialized capillary pH, partial pressure of arterialized capillary O_2_ (PcO_2_) and CO_2_ (PcCO_2_), and arterialized O_2_ saturation (ScO_2_). Standard calibration was performed prior to each blood sample analysis. Arterialized hemoglobin concentration ([Hb]) was measured using an azidemethemoglobin double wavelength photometer method (HemoCue® Hb201+; HemoCue AB, Ängelholm, Sweden). Arterialized capillary O_2_ content (CcO_2_; index of CaO_2_) was calculated using the equation: 1.36 × [Hb] × ScO_2_/100) + 0.003 × PaO_2_.

#### Electromyography

Quadriceps electromyogram (EMG) was recorded from the right vastus lateralis during exercise using electrodes with full-surface solid adhesive hydrogel. Following careful preparation of the skin (shaving, abrading, and cleaning with alcohol) to lower impedance <5 kΩ, pairs of circular silver chloride (recording diameter of 10 mm) surface electrodes (Medi-Trace™ 100; Tyco Healthcare Group, Mansfield, UK) with an interelectrode distance (center-to-center) of 20 mm were placed along the line from the superior lateral side of the patella to the anterior superior iliac spine at ∼100 mm from the patella as described by Rainoldi et al. (2004). The position of the electrodes was marked on the skin for identical placement between exercise sessions. To minimize movement artifacts, the electrodes and cables were secured to the participant's leg using elastic bandage and netting. The EMG signals were amplified (Bio Amp Powerlab 26T; ADInstruments, Bella Vista, Australia) and filtered using a band-pass filter with low-pass cutoff frequency of 10 Hz and a high-pass cutoff frequency of 999 Hz (LabChart version 7.2; ADInstruments). The filtered EMG signals were sampled at 2 kHz by an analog-to-digital converter (PowerLab 26T; ADInstruments). As an index of motor drive, the EMG root mean square (RMS) was calculated for each single muscle contraction (LabChart version 7.2; ADInstruments).

#### Rate of perceived exertion (RPE)

During exercise, the participants were asked to score their RPE on the 0–10 Borg scale every 3 km (Borg 1982). The scale with descriptors was mounted in front of the subject at eye height. At regular intervals they were asked to activate a handle bar mounted switch, activating the appropriate led indicator next to the descriptor corresponding to their RPE, resulting in a corresponding voltage signal being fed into the analog-to-digital converter.

### Data and statistical analysis

Resting values were obtained by averaging the data obtained in the last 30 sec of the 4 min resting period prior to exercise. During the time trial exercise, a single time-weighted mean value for each variable was obtained by averaging the means of the last 20 sec of each km. The effects of hypoxia and CO_2_ clamping on cardiorespiratory, cerebrovascular, and blood gas responses at rest were assessed using a two-way repeated measures ANOVA with α-level of 0.05 (IBM SPSS Statistics version 20.0; IBM Corporation, Armonk, NY). Likewise, we performed two-way repeated measures ANOVA to isolate the effect of hypoxia and CO_2_ clamping on the mean cardiorespiratory, cerebrovascular, and blood gas responses during the 15-km time trial cycling with a α-level of 0.05. Trends were considered when *P* < 0.10. For significant interactions between hypoxia and CO_2_ clamping, four pairwise comparisons (Bonferroni corrected) were performed to isolate the effect of hypoxia and inspired CO_2_ on the dependent measures within participants with a α level of 0.0125, indicated where appropriate with the superscript ^B^. With the exception of RPE, which is reported as median and range, all data are reported as means ± SD.

With the exception of missing data at the end of exercise, which were replaced by repeated imputation using the missing data analysis function from the SPSS program, individual missing data points were replaced by values derived from a linear interpolation procedure.

## Results

All 10 participants completed the experimental protocol. Due to a technical problem, one participant could not complete the final 2 km during the CO_2_ clamping in hypoxia. Accordingly, comparison of exercise time was carried out in nine participants. Due to technical issues, blood sample analysis could not be carried out in all the participants (see Table 2 for details). No participants reported any side effects such as headache or dyspnea following the experiments.

**Table 1:** Effect of hypoxia and CO_2_ clamping on resting respiratory, cerebrovascular, and cardiovascular variables

	Normoxia		Hypoxia	
		
	Control	CO_2_ clamp	Control	CO_2_ clamp
Respiratory				
V'E (L/min)	15.7 ± 2.6	22.3 ± 3.3^1^	16.6 ± 5.9	30.4 ± 4.4^1^,^2^
PETCO_2_ (mmHg)	42 ± 2	45 ± 2^1^	37 ± 1^2^	46 ± 1^1^
PETO_2_ (mmHg)	108 ± 2	118 ± 5^1^	47 ± 2^2^	59 ± 2^1^,^2^
SpO_2_ (%)	97.1 ± 1.6	97.2 ± 1.5	83.5 ± 3.7^2^	89.1 ± 3.1^1^,^2^
Cerebrovascular				
Middle cerebral artery velocity (cm/sec)	60 ± 6	61 ± 7^1^	61 ± 4^2^	69 ± 7^1^,^2^
Cerebral O_2_Hb (▵μmol)	3.3 ± 3.4	3.4 ± 3.9	−1.4 ± 3.9^2^	1.3 ± 2.3^2^
Cerebral HHb (▵μmol)	−0.5 ± 0.7	−0.9 ± 1.8	4.9 ± 2.4^2^	2.3 ± 1.6^1^,^2^
Cerebral DO_2_ (%) *n* = 8	101.7 ± 4.3	106.0 ± 8.3^1^	91.5 ± 8.7	109.3 ± 9.7^1^
Muscle oxygenation				
Muscle O_2_Hb (▵μmol)	2.3 ± 4.1	1.8 ± 6.9	−3.7 ± 4.8^2^	−0.1 ± 5.0^2^
Muscle HHb (▵μmol)	−5.5 ± 2.7	−4.8 ± 3.9	−0.4 ± 6.4^2^	−2.2 ± 3.2^2^
Cardiovascular				
Mean arterial blood pressure (mmHg)	97 ± 6	101 ± 13	100 ± 8	106 ± 11
HR (b/min)	80 ± 10	81 ± 11^1^	92 ± 16^2^	86 ± 10^1^,^2^
Blood gases				
pH	7.46 ± 0.03	7.46 ± 0.05	7.48 ± 0.06	7.46 ± 0.04^1^
PcCO_2_ (mmHg) *n* = 9	34 ± 3	36 ± 3^1^	29 ± 3^2^	37 ± 56^1^
PcO_2_ (mmHg) *n* = 9	98 ± 5	107 ± 8^1^	46 ± 4^2^	57 ± 5^1^,^2^
ScO_2_ (%) *n* = 9	97.9 ± 0.5	98.1 ± 0.3	87.2 ± 4.5^2^	90.9 ± 1.9^1^,^2^
CcO_2_ (mL O_2_/dL) *n* = 9	19.9 ± 1.2	19.9 ± 1.3	17.8 ± 1.7^2^	18.5 ± 1.1^2^

Values are mean±SD. Cerebral and muscle oxygenation data (O_2_Hb and HHb) are expressed as delta change from baseline room air breathing values. Likewise, cerebral DO_2_ is expressed as % of baseline room air breathing values.

1 Different from control (*P* < 0.05).

2 Different from normoxia (*P* < 0.05).

**Table 2:** Effect of hypoxia and CO_2_ clamping on arterialized blood gas variables and cerebral O_2_ delivery during 15-km time trial

	Normoxia		Hypoxia	
		
	Control	CO_2_ clamp	Control	CO_2_ clamp
pH (*n* = 9)				
5 km	7.42 ± 0.08	7.38 ± 0.07	7.40 ± 0.10	7.40 ± 0.06
10 km	7.41 ± 0.07	7.39 ± 0.10	7.41 ± 0.08	7.43 ± 0.04
15 km	7.39 ± 0.06	7.37 ± 0.07	7.39 ± 0.09	7.35 ± 0.07
PcCO_2_ (mmHg) *n* = 9				
5 km	32.0 ± 4.2	34.5 ± 3.2^1^	22.4 ± 2.5^2^	34.5 ± 3.1^1^
10 km	29.6 ± 2.5	33.7 ± 3.2^1^	19.8 ± 4.7^2^	34.2 ± 2.6^1^
15 km	27.6 ± 3.3	33.6 ± 4.1^1^	19.3 ± 3.1^2^	35.1 ± 3.0^1^
PcO_2_ (mmHg) *n* = 8				
5 km	81.2 ± 8.2	86.9 ± 10.7^1^	32.7 ± 4.8^2^	37.7 ± 2.9^1^,^2^
10 km	82.1 ± 9.0	87.2 ± 9.3^1^	34.2 ± 3.3^2^	38.8 ± 3.0^1^,^2^
15 km	82.7 ± 9.7	93.0 ± 17.5^1^	33.6 ± 4.2^2^	39.0 ± 3.8^1^,^2^
ScO_2_ (%) *n* = 7				
5 km	95.4 ± 1.6	95.5 ± 2.2	64.4 ± 5.3^2^	68.6 ± 7.3^1^,^2^
10 km	95.5 ± 1.8	96.4 ± 2.0	65.3 ± 3.4^2^	69.7 ± 6.0^1^,^2^
15 km	95.7 ± 1.8	96.6 ± 2.1	65.8 ± 6.7^2^	68.4 ± 8.5^1^,^2^
CcO_2_ (mL O_2_/dL) *n* = 7				
5 km	19.5 ± 1.3	19.3 ± 1.5	13.1 ± 1.6^2^	14.0 ± 1.0^2^
10 km	19.6 ± 1.5	19.5 ± 1.4	13.3 ± 1.1^2^	14.3 ± 0.8^2^
15 km	19.6 ± 1.2	19.4 ± 1.4	12.7 ± 1.1^2^	14.0 ± 1.4^2^
Cerebral DO_2_ (%) *n* = 7				
5 km	115.5 ± 10.7	122.1 ± 13.2^1^	94.5 ± 9.7^2^	111.8 ± 11.8^1^,^2^
10 km	116.4 ± 11.4	137.8 ± 38.8^1^	102.0 ± 11.1^2^	117.8 ± 9.6^1^,^2^
15 km	117.4 ± 12.9	150.3 ± 37.3^1^	99.5 ± 7.7^2^	122.9 ± 13.6^1^,^2^

Values are mean±SD. Cerebral DO_2_ expressed as % of baseline room air breathing values.

1 Different from control (*P* < 0.05).

2 Different from normoxia (*P* < 0.05).

### Resting variables

#### Respiratory variables

CO_2_ clamping elevated resting V'E during normoxic and hypoxic conditions (*P* < 0.001) (Table 1). Meanwhile, acute hypoxia had a greater effect on V'E during the clamped condition compared to the unclamped condition (interaction: *P* = 0.028). Post hoc analysis showed that acute hypoxia elevated V'E during CO_2_ clamping (*P* = 0.001^B^ vs. normoxia), but not during the unclamped control condition (*P* = 0.210^B^ vs. normoxia). There was an interaction between the effect of hypoxia and CO_2_ clamping on both PETCO_2_ and SpO_2_ (interaction: *P* < 0.001 for both). As such, CO_2_ clamping elevated PETCO_2_ during both normoxic and hypoxic conditions (post hoc: *P* = 0.001^B^ and *P* < 0.001^B^, respectively), whereas acute hypoxia lowered PETCO_2_ during the unclamped control condition (*P* < 0.001^B^) and only tended to lower it during the clamped condition (*P* = 0.098^B^). Post hoc analysis revealed that resting SpO_2_ was lowered by acute hypoxia during both control and clamped conditions (*P* < 0.001^B^ vs. normoxia), whereas CO_2_ clamping selectively elevated SpO_2_ in hypoxia (*P* = 0.002^B^), but not during normoxia (*P* = 0.713^B^). Acute hypoxia lowered resting PETO_2_ (*P* < 0.001), whereas CO_2_ clamping elevated it (*P* < 0.001).

#### Cerebrovascular variables

Both acute exposure to hypoxia and CO_2_ clamping elevated resting MCAv (main effects: *P* = 0.014 and *P* < 0.001, respectively; interaction: *P* = 0.101). Hypoxia at rest lowered cerebral O_2_Hb (*P* = 0.003), whereas CO_2_ clamping had no effect (*P* = 0.222). Hypoxia enhanced the effect of CO_2_ clamping on resting cerebral HHb (interaction: *P* = 0.047). Post hoc tests showed that hypoxia elevated cerebral HHb during control and clamped condition (*P* < 0.001^B^ and *P* = 0.001^B^, respectively), whereas CO_2_ clamp lowered resting cerebral HHb in hypoxia (*P* = 0.001^B^) but not in normoxia (*P* = 0.579^B^). Resting cerebral DO_2_ was unaltered with hypoxia (*P* = 0.227), whereas CO_2_ clamping elevated it (*P* < 0.001) with a trend for this increase to be greater in hypoxia compared to normoxia (interaction: *P* = 0.090). As a result, CO_2_ clamping in hypoxia restored resting cerebral DO_2_ to normoxic values (post hoc: *P* = 0.821^B^ vs. normoxia).

#### Muscle oxygenation

Acute hypoxia lowered resting muscle O_2_Hb and elevated muscle HHb (hypoxia: *P* = 0.024 and *P* = 0.007, respectively), whereas no effect was observed with CO_2_ clamping (clamp: *P* = 0.301 and *P* = 0.602).

#### Cardiovascular variables

Resting HR was higher with acute hypoxia (*P* = 0.001) and lower with CO_2_ clamping (*P* = 0.050, interaction: *P* = 0.085), whereas no differences were observed in MAP with either hypoxia or CO_2_ clamping (main effects: *P* = 0.128 and *P* = 0.136, respectively).

#### Blood gas variables

No differences were observed in resting pH in hypoxia (*P* = 0.508), whereas there was an interaction between the effects of CO_2_ clamping and hypoxia on pH (interaction: *P* = 0.016) despite a lack of effect of CO_2_ clamp per se (*P* = 0.489). Post hoc analysis revealed that pH was lower with CO_2_ clamping in hypoxia (*P* < 0.001^B^ vs. control), but not during normoxia (*P* = 0.112^B^). There was an interaction between hypoxia and CO_2_ clamp on PcCO_2_ (interaction: *P* = 0.018). Post hoc *t*-tests showed that hypoxia lowered PcCO_2_ during the unclamped condition (*P* = 0.010^B^ vs. normoxia) but not during the clamped condition (*P* = 0.630^B^), whereas CO_2_ clamping increased PcCO_2_ in hypoxia (*P* = 0.001^B^ vs. control) but not in normoxia (*P* = 0.035^B^). Meanwhile, both resting PcO_2_ and ScO_2_ were lowered with hypoxia (*P* = 0.001). CO_2_ clamping elevated PcO_2_ during both normoxia and hypoxia (*P* < 0.002) and elevated ScO_2_ in hypoxia (post hoc: *P* = 0.016^B^), but not in normoxia (*P* = 0.115^B^, interaction: *P* = 0.024). Resting CcO_2_ was lower with hypoxia (*P* = 0.010), but unaffected with CO_2_ clamping (*P* = 0.221).

### Time trial

#### Cycling performance

Exercise performance was impaired by 19 ± 7% in hypoxia (1899 ± 69 vs. 1584 ± 62 sec, hypoxia vs. normoxia, *P* < 0.001), while there was a nonsignificant trend for CO_2_ clamping to exert a greater effect on exercise time in hypoxia compared to normoxia (CO_2_: *P* = 0.262, interaction: *P* = 0.097). Nevertheless, post hoc analysis revealed no significant improvement in the exercise time with CO_2_ clamping in hypoxia (1875 ± 72 vs. 1924 ± 5 sec, CO_2_ clamp vs. control; *P* = 0.108^B^) or normoxia (1589 ± 62 vs. 1579 ± 66 sec; *P* = 0.586^B^). Hypoxia lowered mean power, speed, and cadence (*P* < 0.001, *P* < 0.001, and *P* = 0.004, respectively) compared to normoxia, whereas no difference was observed with CO_2_ clamping (*P* = 0.468, *P* = 0.0694, and *P* = 0.656).

#### Rate of perceived exertion

The participants’ RPE during the time trial, as indicated by the Borg score, was higher in hypoxia (hypoxia: *P* = 0.009), but no difference was observed with CO_2_ clamping (clamp: *P* = 0.459).

#### Respiratory variables

**Figure 1:**
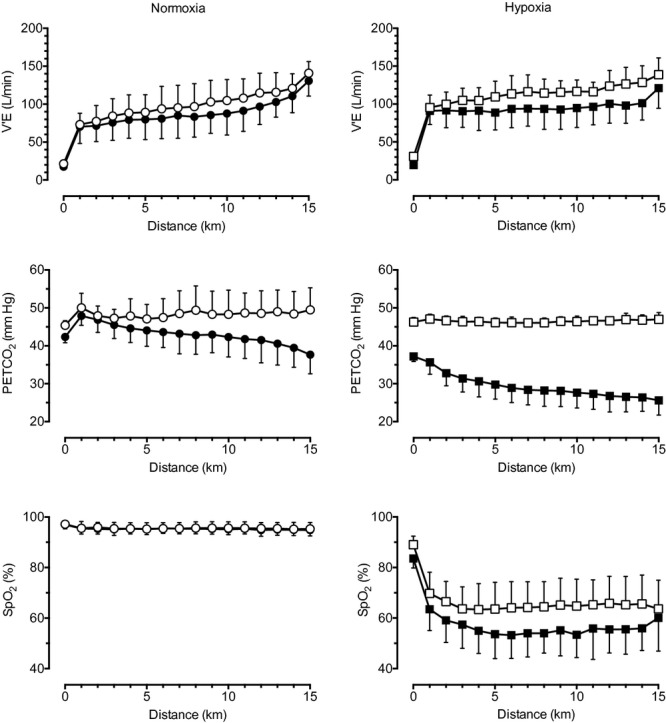
Effect of hypoxia and CO_2_ clamping on respiratory variables during 15-km time trial cycling. Left panels, group data in normoxia (mean ± SD); right panels, group data in hypoxia. •, normoxia control; ○, normoxia CO_2_ clamp; ▪, hypoxia control; □, hypoxia CO_2_ clamp.

V'E was elevated by both hypoxia and CO_2_ clamping (hypoxia: *P* = 0.048, clamp: *P* = 0.023), whereas PETO_2_ was lowered with hypoxia and elevated with CO_2_ clamping throughout the exercise (main effects: *P* < 0.001 for both) (Fig. 1). Meanwhile, PETCO_2_ was lower with hypoxia during control only (*P* < 0.001^B^), whereas CO_2_ clamping restored PETCO_2_ to the values observed in the normoxic clamped condition (*P* = 0.269^B^, interaction: *P* < 0.001). SpO_2_ was lower during exercise in hypoxia during both control and clamped conditions (*P* < 0.001^B^ for both), whereas SpO_2_ was higher with CO_2_ clamping during exercise in hypoxia (*P* < 0.001^B^), but not normoxia (*P* = 0.713^B^, interaction: *P* < 0.001).

In normoxia, the participants began cycling at ∼63% VO_2_max at the start of time trial, which progressively increased to 78% at the 14th km, before a final sprint reaching 85% VO_2_max at the end of exercise. In contrast, during hypoxic conditions, the participants began cycling at 46% (normoxic) VO_2_max, and progressively increased to 57% at the end of the time trial. Overall, both VO_2_ and VCO_2_ were lower in hypoxia (*P* < 0.001 for both), whereas CO_2_ clamping tended to elevate VCO_2_ but not VO_2_ (*P* = 0.064 and *P* = 0.603, respectively).

#### Cerebrovascular variables

**Figure 2:**
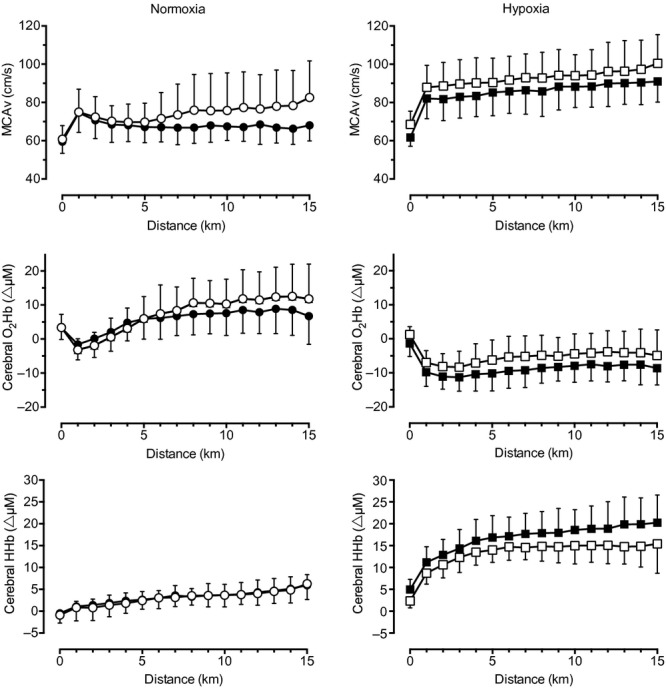
Effect of hypoxia and CO_2_ clamping on cerebral variables during 15-km time trial cycling. Cerebral O_2_Hb and HHb are expressed at delta changes from normoxia (room air baseline). Left panels, group data in normoxia (mean ± SD); right panels, group data in hypoxia. •, normoxia control; ○, normoxia CO_2_ clamp; ▪, hypoxia control; □, hypoxia CO_2_ clamp.

Both hypoxia and CO_2_ clamping elevated MCAv during exercise (by 26% and 9%, respectively; hypoxia: *P* < 0.001, clamp: *P* = 0.024, interaction: *P* = 0.963) (Fig. 2 and Table 1). Hypoxia elevated cerebral HHb during exercise and lowered cerebral O_2_Hb and delta Hb (*P* < 0.001 for all). In contrast, CO_2_ clamping lowered HHb (*P* = 0.015) and elevated delta Hb during exercise (*P* = 0.047), whereas no difference was observed in O_2_Hb with clamping (*P* = 0.118). During the time trial cycling, cerebral DO_2_ was lower with hypoxia (*P* = 0.004) and elevated with CO_2_ clamping (*P* = 0.019, interaction: *P* = 0.911).

#### Cardiovascular variables

There was a nonsignificant trend for MAP to be higher with CO_2_ clamping (*P* = 0.062), whereas no effect was observed with hypoxia (*P* = 0.535). No differences were observed in HR with hypoxia (*P* = 0.206) or CO_2_ clamping (*P* = 0.196).

#### Muscle oxygenation

**Figure 3:**
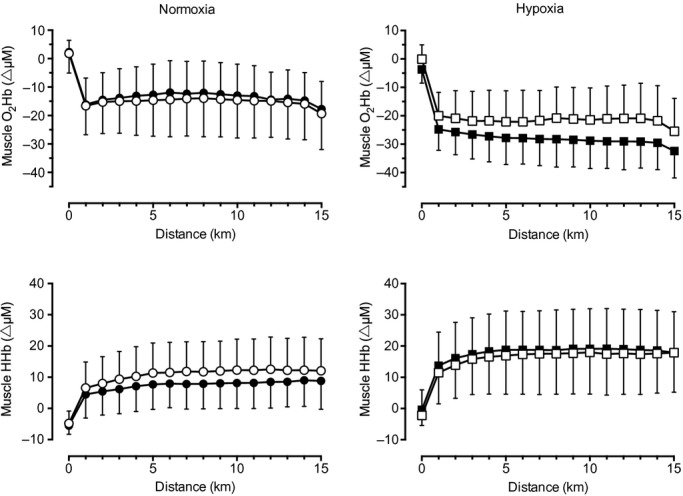
Effect of hypoxia and CO_2_ clamping on muscle oxygenation during 15-km time trial cycling. Muscle O_2_Hb and HHb are expressed at delta changes from normoxia (room air baseline). Left panels, group data in normoxia (mean ± SD); right panels, group data in hypoxia. •, normoxia control; ○, normoxia CO_2_ clamp; ▪, hypoxia control; □, hypoxia CO_2_ clamp.

The effect of CO_2_ clamping on muscle O_2_Hb and delta Hb was greater in hypoxia compared to normoxia (interaction: *P* = 0.039 and *P* = 0.007, respectively) and tended to be greater for muscle HHb (interaction: *P* = 0.078) (Fig. 3). As a result, post hoc analysis revealed that hypoxia lowered muscle O_2_Hb and delta Hb during control condition (*P* < 0.001^B^ vs. normoxia), and by a lesser extend during CO_2_ clamped condition for muscle delta Hb (*P* = 0.002^B^). Meanwhile hypoxia did not alter muscle O_2_Hb in the CO_2_ clamped condition (*P* = 0.033^B^ vs. control). CO_2_ clamping did not alter muscle O_2_Hb in normoxia (*P* = 0.554^B^), but tended to elevate it in hypoxia (*P* = 0.013^B^). Hypoxia during exercise elevated muscle HHb in both control and clamped conditions (*P* = 0.001^B^ and *P* = 0.008^B^ vs. normoxia, respectively), whereas there was a tendency for CO_2_ clamping to elevate muscle HHb during normoxia (*P* = 0.067^B^ vs. control) but not during hypoxia (*P* = 0.460^B^).

During exercise, EMG RMS increased slightly, in parallel with power output, showing a more pronounced increase during the end spurt (*P* < 0.001). EMG RMS was lower in hypoxia compared to normoxic exercise (*P* = 0.002), whereas no difference was observed with CO_2_ clamping (*P* = 0.628).

#### Blood gas variables

CO_2_ clamping during exercise elevated PcCO_2_ by a greater extent in hypoxia compared to normoxia (interaction: *P* < 0.001) (Table 2). Accordingly, post hoc analysis revealed that CO_2_ clamping elevated PcCO_2_ during both normoxia (*P* < 0.001^B^ vs. control) and hypoxia (*P* = 0.004^B^), whereas hypoxia lowered PcCO_2_ during control (*P* = 0.001^B^ vs. normoxia) but no difference was observed between clamped values of PcCO_2_ between normoxia and hypoxia (*P* = 0.526^B^). Hypoxia lowered PcO_2_ and CcO_2_ during exercise (hypoxia: *P* < 0.001 for both), whereas CO_2_ elevated PcO_2_ (*P* < 0.001) and tended to elevate CcO_2_ (*P* = 0.089). The effect of CO_2_ clamping on ScO_2_ was greater in hypoxia than normoxia (interaction: *P* = 0.006). Accordingly, hypoxia lowered ScO_2_ during both control and clamped conditions (post hoc: *P* < 0.001^B^ vs. normoxia), whereas CO_2_ clamp elevated ScO_2_ during hypoxia (*P* = 0.005^B^ vs. control) but not during normoxia (*P* = 0.754^B^). No changes were observed in pH with either hypoxia (*P* = 0.568) or CO_2_ clamping during exercise (*P* = 0.182).

## Discussion

The well-documented detrimental effect of severe hypoxia on exercise performance is not fully understood. Several studies found a relationship between exercise capacity or performance and a reduction in cerebral oxygenation during various exercise modes in hypoxia, such as time trial cycling (Amann 2006; Amann et al. 2006), repeated sprints (Smith and Billaut 2010), incremental exercise (Subudhi et al. 2007a; Peltonen et al. 2009), and static maximal muscle contraction to exhaustion (Rasmussen et al. 2006; Rupp and Perrey 2009; Vogiatzis et al. 2011; Goodall et al. 2012). These observations led to the hypothesis that the hyperventilation-induced hypocapnia and associated reduction in CBF and cerebral oxygenation may account for the impaired capacity and performance in hypoxia.

In this study, we tested the hypothesis that preventing the usual hyperventilation-induced hypocapnia and associated reduction in CBF and cerebral tissue oxygenation would improve 15-km time trial performance in severe normobaric hypoxia. Even though CBF was only increased by 9% during the hypoxic time trial with CO_2_ clamping, it resulted, with the concomitant 9% increase in SpO_2_ (Figs. 2), in significantly increased cerebral DO_2_ by 20% compared to the nonclamped control (Table 2). Importantly, this increase with CO_2_ clamping in hypoxia was sufficient to *normalize* cerebral DO_2_ to levels similar as observed during nonclamped normoxic exercise. As exercise performance in hypoxia was not significantly improved with clamping, it follows that the reduction in overall cerebral O_2_ delivery is not a prime reason for the reduced exercise performance in hypoxia.

### Exercise in hypoxia

The detrimental effects of decreases in CaO_2_ on endurance capacity of large muscle groups are well documented (Adams and Welch 1980; Koskolou and McKenzie 1994; Peltonen et al. 1995; Amann 2006; Amann et al. 2006, 2007). In this study, we found a 9–29% reduction in exercise performance during 15-km time trial cycling in severe hypoxia due to a decreased motor drive (∼16% reduction in right vastus lateralis EMG RMS). In agreement with the results from Amann et al. (2006), we found that markedly different strategies were employed during time trial exercise in normoxia and hypoxia. Specifically, during the time trial in normoxia, the participants slightly increased their power output throughout the 15 km, reaching maximal power at the end of exercise in the form of an end spurt. In contrast, during the time trial in hypoxia, participants began cycling at a relatively high power output, slightly lowering work rate throughout the exercise test, only increasing power output again toward the end with a short sprint over the last 100 m. Interestingly, this progressive decline in central motor drive and power output occurred despite relatively stable CcO_2_, ScO_2_, and muscle [O_2_Hb] during the time trial in hypoxia (Table 2 and Fig. 3). Meanwhile, both cerebral [O_2_Hb] and cerebral DO_2_ remained relatively stable despite parallel increases in MCAv and cerebral [HHb] (Fig. 2 and Table 2). As this decline in power coincided with an increase in perceived exertion, we tentatively interpret these findings as supporting the role of inhibitory feedback of muscle afferents in limiting central motor drive during exercise in severe hypoxia (Amann et al. 2006).

### CO_2_ clamping effects on CBF and prefrontal cerebral oxygenation

Our aim was to improve CBF during exercise by adding CO_2_ to the inspirate_,_ clamping PETCO_2_ to 45 mmHg, preventing the normal hyperventilation-induced hypocapnia and associated cerebral vasoconstriction, while not influencing muscle oxygen supply. As expected, CO_2_ clamping in normoxia prevented the development of hypocapnia during the second half of the time trial, thereby elevating CBF (Fig. 2). During CO_2_ clamping in hypoxia, we were successful in elevating and maintaining PETCO_2_ stable at levels similar to those in normoxia (Fig. 1), without any development of respiratory acidosis (Table 2). However, despite a relatively greater hypercapnic stimulus, we observed only modest elevations in MCAv during exercise with CO_2_ clamping in hypoxia (Fig. 2). In this study, MCAv increased despite relatively stable MAP, cardiac output (not reported), pH, and PcO_2_ during exercise in hypoxia (Fig. 2, Table 2). Therefore, it seems unlikely that changes in perfusion pressure or circulating systemic stimuli (such as arterial hypoxemia) could account for the progressive rise in CBF during time trial cycling observed in hypoxia. Instead, we suspect that the increase in CBF is likely driven by other factors such as increased neural activity and cerebral metabolic demand associated with prolonged aerobic exercise in normobaric hypoxia.

### Control of CBF during hypoxic exercise

The entire capillary network of the brain is covered by extensions of astrocytes, which constitutes the blood–brain barrier (Secher et al. 2007). As such, O_2_ diffusion distance plays a vital role during increased neural activation and subsequent increased O_2_ consumption. As there is no capillary recruitment in the brain, greater CBF is required to elevate the O_2_ gradient (Secher et al. 2007). Acute hypoxic exposure at rest elevates right prefrontal cortex activity (Schneider and Strüder 2009), and enhances O_2_ extraction in the brain (Ho et al. 2008). Meanwhile, studies of dynamically exercising humans have found that the increases in regional MCAv (and CBF) are driven by increased cortical activation of sensorimotor areas associated with neural input from the working limbs (Friedman et al. 1991, 1992; Jorgensen et al. 1992a). These observations support earlier findings in exercising dogs, of CBF redistribution to sensorimotor cerebral cortex, spinal cord, and cerebellum – primarily in cortical layers (Gross et al. 1980). Accordingly, we attribute the higher MCAv observed in this study to greater sensorimotor cortex activation associated with exercise in hypoxia. Despite a considerably lower power output, we observed higher rates of perceived effort during exercise in hypoxia when compared to normoxia, thereby confirming reports of a direct effect of hypoxia on effort perception during exercise in severe hypoxia (Amann et al. 2007). Taken together, we postulate that elevated cerebral metabolism associated with greater sensorimotor cortex activation – as indicated by higher perceived effort – could account for the elevated CBF observed during exercise in severe hypoxia.

### Cerebral oxygenation, oxygen delivery, and performance

Mass cerebral oxygen delivery is the product of CBF and CaO_2_. Therefore, it is important to consider the effect of CO_2_ clamping on CaO_2_. In this study, increased respiratory drive associated with CO_2_ clamping elevated PcO_2_ and ScO_2_, which in term tended to elevate CcO_2_ (*P* = 0.089, Table 2). Combined with modest elevations in MCAv during exercise (Fig. 2), we expected to see elevated mass O_2_ delivery to the brain with CO_2_ clamping in hypoxia. Instead, CO_2_ clamping in hypoxia normalized the cerebral DO_2_ to normoxic control values during time trial cycling (Table 2), which is supported by lower cerebral HHb and higher cerebral delta Hb (Fig. 2). Accordingly, we contend that we were successful in restoring cerebral DO_2_ during the time trial exercise in hypoxia with our CO_2_ clamping setup. Our data corroborate those of Subudhi et al. (2011), who found significant increases in cerebral oxygenation with CO_2_ clamping during incremental exercise to capacity in hypoxia. We also found muscle oxygenation to be elevated with CO_2_ clamping in hypoxia (Fig. 3) – presumably due to a higher SpO_2_ associated with increased ventilation (Fig. 1). As the restoration of cerebral DO_2_ with CO_2_ clamping in our study was not accompanied by improvements in exercise time, motor drive or mean power output, it appears that cerebral deoxygenation associated with hyperventilation-induced hypocapnia cannot account for the impaired performance in hypoxia. Intriguingly, this lack of improvement was observed despite a tendency for improvement of muscle tissue oxygenation with CO_2_ clamping in hypoxia. This lack of effect of increased cerebral O_2_ delivery on performance may be attributed, at least in part, to increased cerebral nonoxidative glycolysis during exercise in hypoxia (Overgaard et al. 2012). Furthermore, it is important to acknowledge that changes in global cerebral DO_2_ may not be representative of changes in local arterial and/or capillary Po_2_ in the brain; therefore, it is possible that we were unable to restore oxygen diffusion drive to the mitochondria of especially active neuronal tissue with our CO_2_ clamping setup.

### Methodological considerations

Some technical considerations should be acknowledged when interpreting our data. Prefrontal lobe oxygenation was used to represent global changes in cerebrocortical oxygenation. Subudhi et al. (2009) have reported good correlations in oxygenation measurements between prefrontal, premotor, and motor cortices during maximal exercise. Therefore, we considered prefrontal oxygenation as an appropriate index of global changes in cerebrocortical oxygenation. Another important issue associated with NIRS is the influence of extracerebral and fat layers on the brain and muscle measurements, respectively (Cooper et al. 2010). Cerebral [O_2_Hb] measurements may be influenced by external carotid blood flow (Li et al. 2011) and forehead skin blood flow (Takahashi et al. 2011) during rest or a verbal fluency task, respectively. Nevertheless, Subudhi et al. (2011) reported significant elevations in cerebral [O_2_Hb] and CBF with CO_2_ clamping during exercise in normoxia and hypoxia. Likewise, we found cerebral [HHb] to be lower with CO_2_ clamping, whereas cerebral [O_2_Hb] tended to be elevated in the last 5 km of exercise during CO_2_ clamping in normoxia and throughout exercise in hypoxia, coinciding with a higher MCAv (Fig. 2). Therefore, we contend that the NIRS methodology used in this study was sensitive enough to detect the changes in cerebral oxygenation associated with CO_2_ clamping.

An important limitation of this study, as of many other studies, is the assumption that MCAv represents global CBF changes. Four lines of evidence support the use of MCAv as an index of global CBF during exercise: (i) MCAv reflects change in internal carotid blood flow during dynamic exercise in both normoxia (Sato et al. 2011) and hypoxia (Huang et al. 1991); (ii) increases in MCAv are matched by increases in cortical CBF during dynamic exercise (Jorgensen et al. 1992a,b); (iii) whereas cortical representation of leg muscle can be served from measuring blood flow velocity of the anterior cerebral artery (van der Zwan and Hillen 1991), cortical representation of muscle involved in cycling is served dominantly by MCA (Jorgensen et al. 1992a); (iv) the cross-sectional area of the MCA remains unchanged within a wide range of changes in PETCO_2_ (Bradac et al. 1976; Giller et al. 1993; Valdueza et al. 1997; Serrador et al. 2000); and (v) the diameter of MCA, as measured using TCD, remains relatively unchanged at 5300 m (5.23 mm) compared to sea level (5.30 mm) (Wilson et al. 2011). Accordingly, we contend that MCAv is a reasonable index of changes in global CBF during heavy exercise in hypoxia. Nevertheless, we acknowledge that changes in CBF and cerebral metabolic activity during exercise and with hypoxia are not homogeneous. In this study, the assessment of CBF and cerebral tissue oxygenation was limited to the MCA and the left prefrontal cortex, respectively, which were taken as global indices. However, it is likely that certain brain regions are more metabolically active and others regions are less active during exercise in hypoxia.

Another limitation of this study is that we clamped end-tidal rather than arterial Pco_2_ during exercise. As the end-tidal–arterial Pco_2_ gradient (Robbins et al. 1990) varies with exercise (Jones et al. 1979; Liu et al. 1995), it is possible that we did not clamp arterial Pco_2_ per se. In this study, we measured arterialized capillary blood samples, as a surrogate of arterial measurements, and were able to clamp and maintain capillary Pco_2_ within 1 mmHg throughout exercise under both normoxic and hypoxic conditions. As Mollard et al. (2010) found good correlations between prewarmed capillary earlobe samples with radial artery measurements of Po_2_ (*R*^2^ = 0.99), Pco_2_ (*R*^2^ = 0.86), and SO_2_ (*R*^2^ = 0.99) during rest, submaximal, and near maximal exercise intensities, we are confident that our protocol was effective in clamping and maintaining arterial Pco_2_.

Finally, lack of power could potentially limit the interpretation of the tendency (*P* = 0.108) for performance to be improved with CO_2_ clamping in hypoxia. However, this seems unlikely as power calculation using Gaussian approximation revealed a power of 0.95 for our post hoc *t*-test. Furthermore, given the results from the earlier studies using an incremental exercise paradigm (Subudhi et al. 2011; Siebenmann et al. 2012) and the rather small effect, if any, found in this study, the tested hypothesis should be refuted.

## Conclusion

We were unable to improve exercise performance in severe hypoxia, despite a normalization of cerebral oxygen delivery to normoxia values and higher cerebral and muscle oxygenation. The hypothesis that the hyperventilation-induced hypocapnia and the subsequent drop in cerebral oxygenation are the cause of the reduced endurance exercise performance in hypoxia is thus refuted. Our data demonstrate that CBF is progressively elevated during time trial cycling in normobaric hypoxia, which appears to be independent of changes in perfusion pressure, cardiac output, or partial pressure of arterial CO_2_ and O_2_. We speculate that this increase in CBF during prolonged submaximal exercise in hypoxia may be due to either greater somatosensory input, increased motor drive output, or both.

## References

[b1] Adams RP, Welch HG (1980). Oxygen uptake, acid-base status, and performance with varied inspired oxygen fractions. J. Appl. Physiol.

[b2] Amann M (2006). Effects of arterial oxygen content on peripheral locomotor muscle fatigue. J. Appl. Physiol.

[b3] Amann M, Dempsey JA (2007). Locomotor muscle fatigue modifies central motor drive in healthy humans and imposes a limitation to exercise performance. J. Physiol.

[b4] Amann M, Kayser B (2009). Nervous system function during exercise in hypoxia. High Alt. Med. Biol.

[b5] Amann M, Eldridge MW, Lovering AT, Stickland MK, Pegelow DF, Dempsey JA (2006). Arterial oxygenation influences central motor output and exercise performance via effects on peripheral locomotor muscle fatigue in humans. J. Physiol.

[b6] Amann M, Romer LM, Subudhi AW, Pegelow DF, Dempsey JA (2007). Severity of arterial hypoxaemia affects the relative contributions of peripheral muscle fatigue to exercise performance in healthy humans. J. Physiol.

[b7] Amann M, Blain GM, Proctor LT, Sebranek JJ, Pegelow DF, Dempsey JA (2011). Implications of group III and IV muscle afferents for high-intensity endurance exercise performance in humans. J. Physiol.

[b60] Borg GA (1982). Psychophysical bases of perceived exertion. Med. Sci. Sports Exerc.

[b8] Bradac GB, Simon RS, Heidsieck CH (1976). Angiographically verified transient alteration of the intracranial arteries and veins in dependence of different CO_2_ tensions. Neuroradiology.

[b9] Calbet JA, Lundby C (2009). Air to muscle O_2_ delivery during exercise at altitude. High Alt. Med. Biol.

[b10] Cohen PJ, Alexander SC, Smith TC, Reivich M, Wollman H (1967). Effects of hypoxia and normocarbia on cerebral blood flow and metabolism in conscious man. J. Appl. Physiol.

[b11] Cooper CE, Penfold SM, Elwell CE, Angus C (2010). Comparison of local adipose tissue content and SRS-derived NIRS muscle oxygenation measurements in 90 individuals. Adv. Exp. Med. Biol.

[b12] Curran LS, Zhuang J, Droma T, Moore LG (1998). Superior exercise performance in lifelong Tibetan residents of 4,400 m compared with Tibetan residents of 3,658 m. Am. J. Phys. Anthropol.

[b13] Currell K, Jeukendrup AE (2008). Validity, reliability and sensitivity of measures of sporting performance. Sports Med.

[b14] Duncan A, Meek JH, Clemence M, Elwell CE, Tyszczuk L, Cope M (1995). Optical pathlength measurements on adult head, calf and forearm and the head of the newborn infant using phase resolved optical spectroscopy. Phys. Med. Biol.

[b61] Fan JL, Kayser B (2013). The effect of adding CO_2_ to hypoxic inspired gas on cerebral blood flow velocity and breathing during incremental exercise. PLoS One.

[b15] Foster C, Green MA, Snyder AC, Thompson NN (1993). Physiological responses during simulated competition. Med. Sci. Sports Exerc.

[b16] Friedman DB, Friberg L, Mitchell JH, Secher NH (1991). Effect of axillary blockade on regional cerebral blood flow during static handgrip. J. Appl. Physiol.

[b17] Friedman DB, Friberg L, Payne G, Mitchell JH, Secher NH (1992). Effects of axillary blockade on regional cerebral blood flow during dynamic hand contractions. J. Appl. Physiol.

[b18] Giller CA, Bowman G, Dyer H, Mootz L, Krippner W (1993). Cerebral arterial diameters during changes in blood pressure and carbon dioxide during craniotomy. Neurosurgery.

[b19] Goodall S, Gonzalez-Alonso J, Ali L, Ross EZ, Romer LM (2012). Supraspinal fatigue after normoxic and hypoxic exercise in humans. J. Physiol.

[b20] Gross PM, Marcus ML, Heistad DD (1980). Regional distribution of cerebral blood flow during exercise in dogs. J. Appl. Physiol.

[b21] Ho Y-CL, Vidyasagar R, Shen Y, Balanos GM, Golay X, Kauppinen RA (2008). The BOLD response and vascular reactivity during visual stimulation in the presence of hypoxic hypoxia. NeuroImage.

[b22] Huang SY, Tawney KW, Bender PR, Groves BM, McCullough RE, McCullough RG (1991). Internal carotid flow velocity with exercise before and after acclimatization to 4,300 m. J. Appl. Physiol.

[b23] Ide K, Horn A, Secher NH (1999). Cerebral metabolic response to submaximal exercise. J. Appl. Physiol.

[b24] Jeukendrup AE, Hopkins S, Aragón-Vargas LF, Hulston C (2008). No effect of carbohydrate feeding on 16 km cycling time trial performance. Eur. J. Appl. Physiol.

[b25] Jones NL, Robertson DG, Kane JW (1979). Difference between end-tidal and arterial PCO_2_ in exercise. J. Appl. Physiol.

[b26] Jorgensen LG, Perko G, Secher NH (1992a). Regional cerebral artery mean flow velocity and blood flow during dynamic exercise in humans. J. Appl. Physiol.

[b27] Jorgensen LG, Perko M, Hanel B, Schroeder TV, Secher NH (1992b). Middle cerebral artery flow velocity and blood flow during exercise and muscle ischemia in humans. J. Appl. Physiol.

[b28] Kjaer M, Hanel B, Worm L, Perko G, Lewis SF, Sahlin K (1999). Cardiovascular and neuroendocrine responses to exercise in hypoxia during impaired neural feedback from muscle. Am. J. Physiol.

[b29] Koskolou MD, McKenzie DC (1994). Arterial hypoxemia and performance during intense exercise. Eur. J. Appl. Physiol. Occup. Physiol.

[b30] Li Z, Zhang M, Xin Q, Li J, Chen G, Liu F (2011). Correlation analysis between prefrontal oxygenation oscillations and cerebral artery hemodynamics in humans. Microvasc. Res.

[b31] Liu Z, Vargas F, Stansbury D, Sasse SA, Light RW (1995). Comparison of the end-tidal arterial PCO_2_ gradient during exercise in normal subjects and in patients with severe COPD. Chest.

[b32] Madsen PL, Sperling BK, Warming T, Schmidt JF, Secher NH, Wildschiodtz G (1993). Middle cerebral artery blood velocity and cerebral blood flow and O_2_ uptake during dynamic exercise. J. Appl. Physiol.

[b33] Mollard P, Bourdillon N, Letournel M, Herman H, Gibert S, Pichon A (2010). Validity of arterialized earlobe blood gases at rest and exercise in normoxia and hypoxia. Respir. Physiol. Neurobiol.

[b34] Nybo L, Rasmussen P (2007). Inadequate cerebral oxygen delivery and central fatigue during strenuous exercise. Exerc. Sport Sci. Rev.

[b35] Overgaard M, Rasmussen P, Bohm AM, Seifert T, Brassard P, Zaar M (2012). Hypoxia and exercise provoke both lactate release and lactate oxidation by the human brain. FASEB J.

[b36] Palmer GS, Dennis SC, Noakes TD, Hawley JA (1996). Assessment of the reproducibility of performance testing on an air-braked cycle ergometer. Int. J. Sports Med.

[b37] Peltonen JE, Rantamaki J, Niittymaki SP, Sweins K, Viitasalo JT, Rusko HK (1995). Effects of oxygen fraction in inspired air on rowing performance. Med. Sci. Sports Exerc.

[b38] Peltonen JE, Paterson DH, Shoemaker JK, DeLorey DS, duManoir GR, Petrella RJ (2009). Cerebral and muscle deoxygenation, hypoxic ventilatory chemosensitivity and cerebrovascular responsiveness during incremental exercise. Respir. Physiol. Neurobiol.

[b39] Rainoldi A, Melchiorri G, Caruso I (2004). A method for positioning electrodes during surface EMG recordings in lower limb muscles. J. Neurosci. Methods.

[b40] Rasmussen P, Dawson EA, Nybo L, Secher JJ, van Lieshout NH, Gjedde A (2006). Capillary-oxygenation-level-dependent near-infrared spectrometry in frontal lobe of humans. J. Cereb. Blood Flow Metab.

[b41] Rasmussen P, Nielsen J, Overgaard M, Krogh-Madsen R, Gjedde A, Secher NH (2010). Reduced muscle activation during exercise related to brain oxygenation and metabolism in humans. J. Physiol.

[b42] Robbins PA, Conway J, Cunningham DA, Khamnei S, Paterson DJ (1990). A comparison of indirect methods for continuous estimation of arterial PCO_2_ in men. J. Appl. Physiol.

[b43] Rupp T, Perrey S (2009). Effect of severe hypoxia on prefrontal cortex and muscle oxygenation responses at rest and during exhaustive exercise. Adv. Exp. Med. Biol.

[b44] Sato K, Ogoh S, Hirasawa A, Oue A, Sadamoto T (2011). The distribution of blood flow in the carotid and vertebral arteries during dynamic exercise in humans. J. Physiol.

[b45] Schneider S, Strüder HK (2009). Monitoring effects of acute hypoxia on brain cortical activity by using electromagnetic tomography. Behav. Brain Res.

[b46] Secher NH, Seifert T, Van Lieshout JJ (2007). Cerebral blood flow and metabolism during exercise: implications for fatigue. J. Appl. Physiol.

[b47] Serrador JM, Picot PA, Rutt BK, Shoemaker JK, Bondar RL (2000). MRI measures of middle cerebral artery diameter in conscious humans during simulated orthostasis. Stroke.

[b48] Siebenmann C, Sørensen H, Jacobs RA, Haider T, Rasmussen P, Lundby C (2012). Hypocapnia during hypoxic exercise and its impact on cerebral oxygenation, ventilation and maximal whole body O_2_ uptake. Respir. Physiol. Neurobiol.

[b49] Smith KJ, Billaut F (2010). Influence of cerebral and muscle oxygenation on repeated-sprint ability. Eur. J. Appl. Physiol.

[b50] Subudhi AW, Dimmen AC, Roach RC (2007a). Effects of acute hypoxia on cerebral and muscle oxygenation during incremental exercise. J. Appl. Physiol.

[b51] Subudhi AW, Lorenz MC, Fulco CS, Roach RC (2007b). Cerebrovascular responses to incremental exercise during hypobaric hypoxia: effect of oxygenation on maximal perfor-mance. Am. J. Physiol. Heart Circ. Physiol.

[b52] Subudhi AW, Miramon BR, Granger ME, Roach RC (2009). Frontal and motor cortex oxygenation during maximal exercise in normoxia and hypoxia. J. Appl. Physiol.

[b53] Subudhi AW, Olin JT, Dimmen AC, Polaner DM, Kayser B, Roach RC (2011). Does cerebral oxygen delivery limit incremental exercise performance?. J. Appl. Physiol.

[b54] Takahashi T, Takikawa Y, Kawagoe R, Shibuya S, Iwano T, Kitazawa S (2011). Influence of skin blood flow on near-infrared spectroscopy signals measured on the forehead during a verbal fluency task. NeuroImage.

[b55] Valdueza JM, Balzer JO, Villringer A, Vogl TJ, Kutter R, Einhaupl KM (1997). Changes in blood flow velocity and diameter of the middle cerebral artery during hyperventilation: assessment with MR and transcranial Doppler sonography. AJNR.

[b56] Verges S, Rupp T, Jubeau M, Wuyam B, Esteve F, Levy P (2012). Cerebral perturbations during exercise in hypoxia. Am. J. Physiol. Regul. Integr. Comp. Physiol.

[b57] Vogiatzis I, Louvaris Z, Habazettl H, Athanasopoulos D, Andrianopoulos V, Cherouveim E (2011). Frontal cerebral cortex blood flow, oxygen delivery and oxygenation during normoxic and hypoxic exercise in athletes. J. Physiol.

[b58] Wilson MH, Edsell MEG, Davagnanam I, Hirani SP, Martin DS, Levett DZH (2011). Cerebral artery dilatation maintains cerebral oxygenation at extreme altitude and in acute hypoxia; an ultrasound and MRI study. J. Cereb. Blood Flow Metab.

[b59] van der Zwan A, Hillen B (1991). Review of the variability of the territories of the major cerebral arteries. Stroke.

